# Single-Stage Debridement and Spinal Instrumentation for Salmonella Spondylodiscitis of Thoracic Vertebra

**DOI:** 10.7759/cureus.18306

**Published:** 2021-09-26

**Authors:** Muhammad Zaki, Mohd Hisam Muhammad Ariffin

**Affiliations:** 1 Orthopaedics and Traumatology, Universiti Kebangsaan Malaysia Medical Centre, Kuala Lumpur, MYS; 2 Spine Surgery, Universiti Kebangsaan Malaysia Medical Centre, Kuala Lumpur, MYS

**Keywords:** salmonella infection, spondylodiscitis, thoracic spine, thoracic spine infection, spine instrumentation

## Abstract

For patients with pyogenic spondylodiscitis, medical therapy with antibiotics is the first line of treatment. Response to antibiotics can be assessed by improvement of symptoms, reduction in inflammatory markers, and radiological evidence of infection resolution with magnetic resonance imaging (MRI). We report a case of a 60-year-old man who presented with intermittent fever and persistent back pain. He was initially treated with an intravenous antibiotic for sepsis secondary to Salmonella enteritidis bacteremia. His spine MRI showed compression of the T10 vertebra with vertebral abnormality suggestive of metastases. He showed no clinical improvement after a course of intravenous antibiotics. Following that, debridement and spinal instrumentation of the thoracic vertebra were done in single-stage surgery.

## Introduction

Pyogenic spondylodiscitis caused by Salmonella species is rare, as the organism usually manifests infection in the intestinal tract. There were two reported cases of Salmonella spondylodiscitis in Malaysia, and both were successfully treated with a course of antibiotics [1,2]. Mazlan et al. reported that their patient was successfully treated with intravenous ampicillin 2 g for two weeks, followed by oral ciprofloxacin 750 mg for three months [1]. Another case reported the use of ceftriaxone for two months in the treatment of Salmonella spondylodiscitis patient [2]. We report a case of Salmonella enteritidis spondylodiscitis that shows no clinical improvement after a course of antibiotics. Surgical debridement was performed to diagnose and eradicate the infection. Following debridement, spinal instrumentation was performed in a single-stage surgery.

## Case presentation

**Figure 1:**
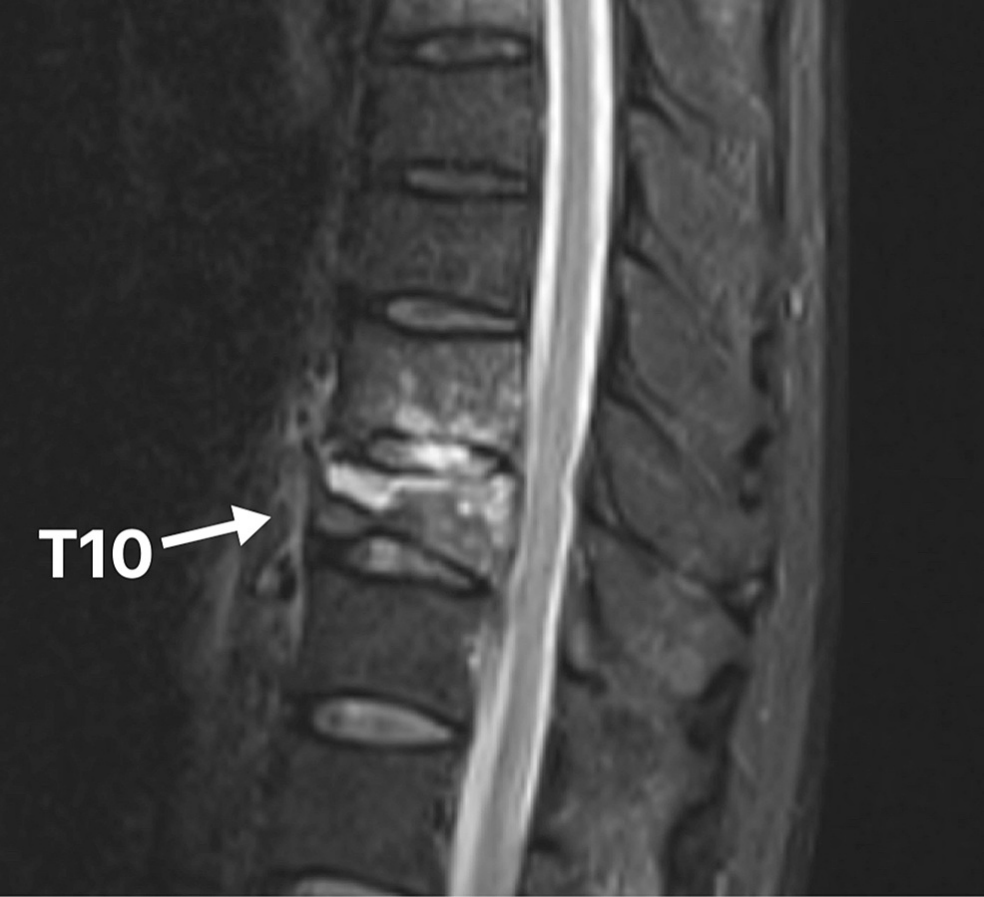
MRI spine sagittal view revealed hyperintense lesion over T9 and T10 vertebral bodies.

A 60-year-old man with underlying anti-neutrophil cytoplasmic antibody (ANCA) negative vasculitis presented with intermittent fever and progressive back pain for two weeks duration. There was no history of trauma, diarrhea, chronic cough, or hemoptysis. He denied any contact with persons afflicted with tuberculosis. On presentation, he was alert and orientated to time, place, and person. He was able to walk with a normal gait. Examination revealed spinal tenderness over the thoracolumbar junction. The neurological status of his upper and lower limbs was intact. Other systemic reviews were normal. The inflammatory markers were elevated; erythrocyte sedimentation rate (ESR) was 120 mm/hr and C-reactive protein (CRP) was 31 mg/dl. Mantoux test was negative. Thoracolumbar radiograph revealed a compression fracture of T10. He was initially treated in the medical ward for sepsis secondary to Salmonella enteritidis bacteremia. Initially, he was given ceftriaxone; however, it was discontinued as the patient developed an allergic reaction to the antibiotic. He was then given intravenous meropenem for one week. Subsequently, fever subsided; however, the back pain was worse than before. The pain was aggravated when he tries to sit up or rotate his body. Otherwise, he had no weakness or numbness of limbs. He also did not have urinary or bowel incontinence. Further imaging with MRI of the whole spine showed a compression fracture of the T10 vertebra with vertebral abnormality suggestive of infection or malignancy (Figure 1). Medical therapy with IV amoxicillin-clavulanic acids was commenced for two weeks. However, the back pain still persisted, and the blood inflammatory markers trend showed no improvement (Table 1). He also developed autoimmune hemolytic anemia secondary to the infection. He was then planned for surgical debridement and fixation of his spine in single-stage surgery.

**Table 1:** Inflammatory markers trend. ESR, erythrocyte sedimentation rate; CRP, C-reactive protein.

Date	16/12/2019	12/1/2020	16/1/2020	23/1/2020	(Post-op)
ESR (mm/hr)	>120	101	-	>120	98
CRP (mg/dl)	31	21	28	27	12

**Figure 2:**
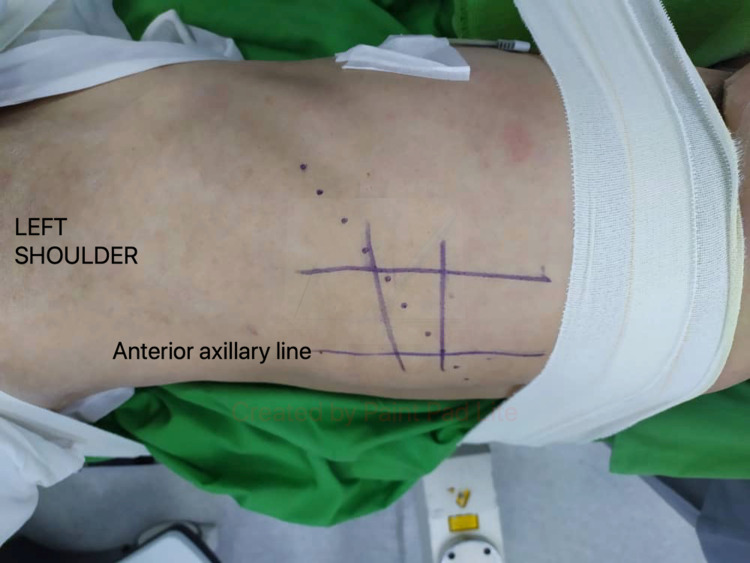
Surgical incision marking (dotted line) for transthoracic approach.

**Figure 3:**
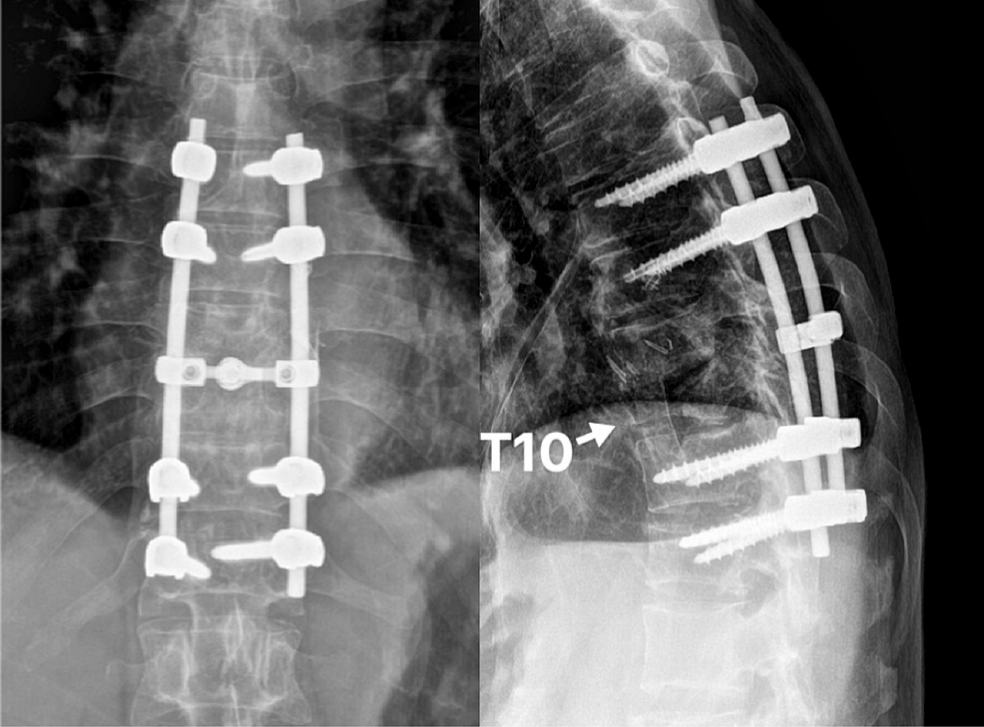
Postoperative spine radiograph.

Surgery was done under general anesthesia, and the patient was continuously monitored using the neuro-electrophysiological method. The patient was positioned right lateral decubitus on the operation table. The patient’s body was supported with rolled cloth and secured to the operative table by using adhesive tape. His legs were flexed and separated with a pillow. The level of the vertebra was identified with image intensifier guidance. A skin incision was made over the intercostal space, starting from the level of the anterior axillary line and curved posteriorly (Figure 2). The periosteum of the rib was carefully divided, and the segment of the rib was resected. The rib segment was resected to allow a direct approach to the T10 vertebra, and it was also used as a strut graft later. Once the thorax was opened, the lungs were deflated and retracted. The parietal pleura was incised to expose the vertebral body. The intraoperative finding revealed a collection of pus within the T10 vertebra and spreading along the adjacent anterior longitudinal ligament. Devitalized disc and bone were meticulously debrided, and curettage was done to the healthy boundary. The area of surgery was then extensively irrigated with normal saline. An autologous bone graft of appropriate length (utilizing resected rib segment) was packed to the vertebral defect to reconstruct the anterior column. A chest tube was inserted, and the wound was closed in layers. The patient was then turned to the prone position. A midline incision was made and the wound was opened in layers until posterior elements of the spine were exposed. Then posterior instrumentation of T8-T9 and T12-L1 was done in the prone position. Post-operative spine radiograph showed good screws placement and acceptable spinal alignment (Figure 3).

Postoperatively, the patient showed recovery in back pain. He was given a course of azithromycin, as suggested by an infectious disease consultant. Intraoperative bone and tissue culture revealed Salmonella enteritidis, which is sensitive to ceftriaxone and ampicillin. The tissue for tuberculosis polymerase chain reaction was negative. Tissue for histopathological examination showed acute/chronic inflammation changes and no evidence of granulomatous inflammation or malignancy. The patient was able to ambulate using a walking frame prior to discharge.

## Discussion

Spinal infection can be classified aetiologically as pyogenic, granulomatous (tuberculosis, brucellosis, or fungal infection), or parasitic. Medical therapy is usually the basis of treatment for pyogenic spondylodiscitis. According to the National Antibiotic Guideline, empirical antibiotic therapy for pyogenic spondylodiscitis should be withheld before a biopsy is performed, unless the patient is in sepsis [3]. An empirical antibiotic cloxacillin can then be initiated, and the antibiotic can be changed later according to the microbial culture sensitivity [3]. Duration of antibiotics is a minimum for six weeks, or eight weeks if an undrained paravertebral abscess or infection due to a drug-resistant organism is found [3].

Salmonella is gram-negative bacilli that can cause infection commonly in the gastrointestinal tract. Salmonella infection may be started by ingestion of the organism from contaminated water or food. Symptoms of acute infection such as diarrhea, vomiting, or abdominal pain usually can be treated conservatively. However, Salmonella can persistently exist in host cells causing chronic infection. Commonly used antibiotics to treat Salmonella infections are ceftriaxone and ampicillin [4]. In our case, the patient initially presented with sepsis with no obvious source of infection. Blood culture revealed that he had Salmonella enteritidis bacteremia. MRI was done in view of persistent back pain and static inflammatory markers despite a course of antibiotics, which revealed possible spine infection or malignancy. He may have chronic Salmonella infection because of his immuno-compromise status (he was on prednisolone for his vasculitis), which was then spread to his spine. However, he denies a history of a recent diarrhoeal disease, which may indicate acute Salmonella infection.

The purposes of surgery in the treatment of spondylodiscitis are for debridement of the infection, collection of tissue for bacterial culture and histopathological examination, decompression of spinal canal, and stabilization of spinal segment. Surgical intervention is indicated when patients develop sepsis, or significant neurological deficit, or show no response to medical therapy [5]. Other relative indications include uncontrollable pain, impending spine deformity, or the patient’s lack of compliance with medication [5].

Surgical debridement for our patient was performed utilizing the transthoracic approach. The transthoracic approach allowed safe and radical debridement of the infected spinal structure. After radical debridement and irrigation, our patient proceeded with spinal fixation in the same setting. Study shows that debridement and spine instrumentation can be performed in a single-stage surgery for spine infection [6]. The advantages are early post-operative rehabilitation, reduced risk of multiple surgeries, and shorter hospital stay [6]. We decided to proceed with spine instrumentation in the same setting because healthy boundaries were achieved during the radical debridement. However, implantation of metal near the infectious lesion may carry the risk of recurrence of the infection later. We used the resected rib segment to fill in vertebral defect at the anterior column as it can provide structural support.

## Conclusions

In conclusion, medical therapy with antibiotics is the first-line treatment for pyogenic spondylodiscitis; however, surgical debridement with spinal fixation needs to be considered if indicated. Single-stage surgery of spinal debridement and spinal fixation can be performed for patients with pyogenic spondylodiscitis. These will benefit patients with multiple medical comorbidities as they can avoid the risk of multiple surgeries.
